# Epidemiological Overview of African Swine Fever in Uganda (2001–2012)

**DOI:** 10.1155/2013/949638

**Published:** 2013-08-12

**Authors:** David Kalenzi Atuhaire, Sylvester Ochwo, Mathias Afayoa, Frank Norbert Mwiine, Ikwap Kokas, Eugene Arinaitwe, Rose Anna Ademun-Okurut, Julius Boniface Okuni, Ann Nanteza, Christosom Ayebazibwe, Loyce Okedi, William Olaho-Mukani, Lonzy Ojok

**Affiliations:** ^1^College of Veterinary Medicine, Animal Resources and Biosecurity, Makerere University, P.O. Box 7062, Kampala, Uganda; ^2^National Agricultural Research Organization, National Livestock Resources Research Institute, P.O. Box 96, Tororo, Uganda; ^3^Ministry of Agriculture, Animal Industry and Fisheries, National Animal Disease Diagnostics and Epidemiology Centre, P.O. Box 513, Entebbe, Uganda; ^4^African Union-Inter African Bureau for Animal Resources, P.O. Box 30786, Nairobi, Kenya

## Abstract

African swine fever (ASF) is a contagious viral disease, which can cause up to 100% mortality among domestic pigs. In Uganda there is paucity of information on the epidemiology of the disease, hence a study was carried out to elucidate the patterns of ASF outbreaks. Spatial and temporal analyses were performed with data collected monthly by the district veterinary officers (DVOs) and sent to the central administration at MAAIF from 2001 to 2012. Additionally, risk factors and the associated characteristics related to the disease were assessed based on semistructured questionnaires sent to the DVOs. A total of 388 ASF outbreaks were reported in 59 districts. Of these outbreaks, 201 (51.8%) were reported in districts adjacent to the national parks while 80 (20.6%) were adjacent to international borders. The number of reported ASF outbreaks changed over time and by geographical regions; however, no outbreak was reported in the North-Eastern region. ASF was ranked as second most important disease of pigs, and it occurred mostly during the dry season (*P* = 0.01). Pig movements due to trade (OR 15.5, CI 4.9–49.1) and restocking (OR 6.6, CI 2.5–17.3) were the major risk factors. ASF control strategies should focus on limiting pig movements in Uganda.

## 1. Introduction

African swine fever (ASF) is a highly fatal disease of domestic pigs and can cause mortality of up to 100% of affected pigs [1]. The disease is caused by double-stranded DNA virus with an icosahedral symmetry that belongs to genus *Asfivirus* and family *Asfarviridae* [2]. Since its first description in Kenya in the early 1920s [3], the disease has been reported in several countries around the world, remaining endemic in Sardinia, and in 2007 outbreaks was reported in Georgia, Russia, and neighbouring countries [4]. The epidemiology of ASF is complex, transmission is direct and vector-borne, and the disease has well-recognized sylvatic and domestic cycles. In sub-Saharan Africa, ASFV is maintained by long-term, inapparent infection of wildlife hosts such as bush pigs (*Potamochoerus porcus*) and warthogs (*Phacochoerus africanus*) which are infected via tick bites of the argasid tick vector (*Ornithodoros *complex) [5].

ASF is highly contagious and is transmitted by direct contact between infected pigs and susceptible ones or by contact with or ingestion of infectious secretions/excretions. The virus is highly resistant in tissues and the environment, contributing to its transmission over long distances through swill feeding and fomites (e.g., contaminated material, vehicles, or visitors to pig premises) [6]. In subacute cases pigs lose condition and die of pneumonia. Chronically, survivors are characterized by emaciation, stunted growth, hemorrhagic necrosis of the skin overlying bony protuberances, followed by abscessation, and deep ulceration. Acute disease caused by the virus is characterized by high fever, hemorrhages in the reticuloendothelial system, and high morbidity and mortality rates with consequent economic losses [7]. Unlike domestic swine, ASFV infections of wild swine are asymptomatic with low viraemia titers [8]. The wild swine and soft ticks of the genus *Ornithodoros *act as a virus reservoir [5]. This large natural reservoir of virus poses a constant threat to domestic pig populations worldwide.

Pig farming is one of the fastest growing livestock activities in the rural areas of Uganda and has become very attractive through the country as a means of increasing food, but income and employment have on several occasions been hampered by ASF [9]. According to reports, Uganda has the largest and fastest growing pig production in Eastern Africa with the pig population standing at 3.2 million [10]. But ASF is an economically important and frequently lethal disease of domestic pigs which has hampered the development of the pig industry. The aim of the study was to elucidate the patterns of ASF outbreaks in Uganda based on the spatial and temporal retrospective data retrieved from monthly reports from district veterinary officers (DVOs) to the central administration at the Ministry of Agriculture, Animal Industry and Fisheries (MAAIF) for the years spanning 2001–2012 to give an insight in the epidemiology of the disease in Uganda.

## 2. Materials and Methods

Retrospective data on ASF outbreaks in Uganda during 2001–2012 were retrieved from MAAIF, Uganda. The information was based on the monthly ASF disease surveillance reports from the DVOs. For the period considered under this study, Uganda had a differing number of districts (80–120). The lowest administrative units reporting ASF outbreaks in Uganda are districts, and, in this work, these were used as epidemiological units of ASF outbreaks and classified as adjacent to the national park(s) and international border(s). The national parks considered in this study included: Queen Elizabeth National Park (QENP), Lake Mburo National Park (LMNP), Murchison Falls National Park (MFNP), Kidepo Valley National Park (KVNP), Rwenzori Mountains National Park (RMNP), Kibale National Park (KINP), Mount Elgon National Park (MENP), Bwindi Impenetrable National Park (BINP), Mgahinga Gorilla National Park (MGNP), and Semuliki National Park (SNP).

Rainfall values and the seasons for the different months, districts, and years (2001–2008) were obtained from the Meteorological Department, Ministry of Energy, Water and Mineral Resources, Uganda. For convenience of data handling and presentation, seven geographical regions, which in most cases vary by rainfall [11], agroecology, and farming production systems, were considered. The months of the year were categorized as having below average rainfall (dry season), above average rainfall (wet season), and average rainfall (neither wet nor dry season) based on long term mean monthly rainfall over the period 2001–2008. A nonparametric test (Kruskal-Wallis rank test) was used to assess the effect of season on ASF (Stata 12.0). ArcGIS (version 10) was used to plot the district distribution of ASF outbreaks (2001–2012).

Semistructured questionnaires on the occurrence of ASF outbreaks and the perception of risk factors and characteristics of these outbreaks were administered to the DVOs in their respective districts. General subject introductions and clarifications were made immediately after the distribution of the questionnaires. Questions included whether there are cases of pig deaths or sickness, percentage of the pig population affected, rating of ASF as an important disease, seasons when incidence is most frequent, and actions taken to control ASF. Questions were answered by ticking prewritten choices, whilst additional information was supplied in the extra spaces provided. Opinions and data were collected, entered into Excel 2010, summarized, coded, and analyzed. Perceived risk factors were scored on a Likert scale [12] and categorized as less or not important (below 25%), important (26–100%), or not applicable so as to estimate the corresponding odds ratios (ORs) as described previously [13]. Both descriptive statistics (contingency tables) and inferential statistics such as confidence interval for odds ratio were computed so as to quantify the major reason for pig movement in the area.

## 3. Results

### 3.1. Temporal Patterns for the Occurrence of ASF

The regional monthly reports of ASF outbreaks from districts during the years 2001–2012 are summarized in Table 1. The total number of districts which reported ASF was 59 with a total of 388 outbreaks. The number of reported ASF outbreaks was highest (68) in 2011 and lowest (6) in 2009. More outbreaks were reported during the months of February (43), March (42), June (40), and January (38) than during the remaining months of the year. The months of October (19), May (20), and September (26) had the lowest number of outbreaks reported. In the districts and regions where rainfall data were available (Table 2), it was apparent that the occurrence of ASF was significantly associated with dry season (*P* = 0.01), when mean monthly rainfall (mm) was below average and the times when animal movement are more frequent.

### 3.2. Spatial Patterns for the Occurrence of ASF

#### 3.2.1. Regional Occurrence of ASF Outbreaks

Regional occurrence of ASF outbreaks is summarized in Table 3. The Central region reported the highest number of ASF outbreaks (181) followed by the Eastern (100), Northern (60), Western (23), Southwestern (12), and West Nile (12). However, there was no ASF outbreak reported in the Northeastern region throughout the period covered by the study. It should be noted that outbreaks were sporadic in the Southwest and West Nile regions throughout the study period.

#### 3.2.2. Occurrence of ASF Outbreaks Adjacent to National Parks

A total of 201 outbreaks were reported in districts adjacent to the national parks representing 51.8% of the total outbreaks reported during the period (2001–2012) as summarized in Table 4.

#### 3.2.3. Occurrence of ASF Outbreaks Adjacent to the International Borders

**Figure 1:**
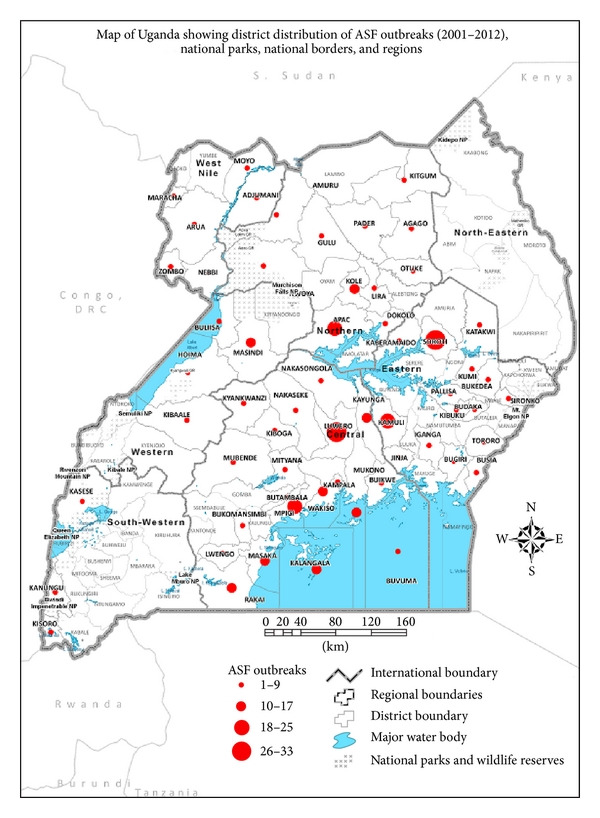
Map of Uganda showing distribution of ASF outbreaks (2001-2012), national parks, national borders, and regions.

Uganda is landlocked and shares borders with Southern Sudan (435 km) on the northern side, Democratic Republic of Congo (DRC, 765 km) on the western side, Tanzania (396 km) and Rwanda (169 km) on the southern side, and Kenya (933 km) on the eastern side (Figure 1). The total number of districts adjacent to the international borders reporting ASF outbreaks was 18. Eighty (20.6%) ASF outbreaks occurred in 18 districts along the international borders compared to 308 outbreaks that occurred in districts that did not share an international border. The number of ASF outbreaks varied between the different international borders, the highest being adjacent with DRC (31 outbreaks in eight districts) and Tanzania borders (26 outbreaks in 2 districts) while only 3 districts bordering Kenya reported 13 outbreaks. The lowest number of ASF outbreaks was reported among the districts bordering Rwanda (one outbreak in one district) and Southern Sudan (9 outbreaks in 4 districts).

#### 3.2.4. Responses of DVOs to the Semistructured Questionnaire about ASF Occurrence in Uganda

Opinions among the DVOs about the characteristics of occurrence and control of ASF in Uganda are summarized in Table 5. In all the 29 districts visited, all the DVOs reported that farmers valued pigs in their areas and that pig farming ranked third in relation to all the other livestock.

ASF was ranked as the second most important disease of pigs after helminthiasis. When faced with problems of pig health, all DVOs reported that they diagnose by use of clinical signs, and only 15 combined the use of clinical signs and laboratory testing. It was however established that the majority of laboratory testing is done at the National Disease Diagnostics and Epidemiological Center in Entebbe with few sending samples to the College of Veterinary Medicine, Makerere University. Twenty-five (86.2%) and 22 (75.9%) of the DVOs reported that the source of ASF outbreaks was as a result of pig movements due to trade (odds ratio OR 15.5, confidence interval CI 4.9–49.1) and pig restocking (odds ratio OR 6.6, confidence interval CI 2.5–17.3), respectively. However, the majority of the DVOs blamed a neighbouring district as a source of their own outbreaks. Only 2 DVOs attributed the outbreaks to wild pigs. Only 1 DVO (Kasese) reported a source of a former outbreak as from a neighbouring country (DRC).

## 4. Discussion

This study aimed at elucidating the patterns of African swine fever outbreaks in Uganda based on the spatial and temporal retrospective data retrieved from monthly reports from DVOs. The study also aimed at describing the perceptions of DVOs on the characteristics of ASF outbreaks in their areas by use of a questionnaire. ASF outbreaks occurred during the entire period of study (2001–2012). Our research findings have proved that ASF is endemic in Uganda since throughout the study period ASF was reported. These findings agree with a previous study in Uganda [14] (unpublished data). A report by the OIE has also indicated that ASF is an endemic disease in Uganda [15].

The distribution of ASF outbreaks showed no specific patterns; however, there was a significant difference in the regions on reported ASF outbreaks with the central region being the most affected (181 outbreaks), and yet it has the highest pig population [10]. Consumption of pork has increased in Uganda with the central region leading in demand. This could be the reason leading to a surge in movement of pigs by traders from most parts of the country to the central region, hence the highest number of outbreaks. The Eastern region reported 100 outbreaks followed by Northern (60) and Western (23) with Southwestern and West Nile reporting only 12 outbreaks each. However, there was no ASF outbreak reported in the North-Eastern region. This difference could be partly because of the proximity to the central administration at MAAIF, methods used in reporting, misdiagnosis, the different husbandry practices, and vigilance in disease control, animal movements, distribution of pig markets, and other virus transmission dynamics. In the North-Eastern region the majority of the population being majorly pastoralists could be the reason that no ASF was reported in that region. More so, the North-Eastern region has the lowest number of pigs and the lowest region household average in Uganda [10].

In the present study, there was a tendency to a seasonal pattern with higher frequency of ASF outbreaks reported during the months with lower or without rainfall. Rainfall distribution in Uganda has been shown to follow 14 distinct climate zones, which often span beyond the administrative partitions [11]. An exact analysis of time-specific rainfall data from a smaller number of districts confirmed that outbreaks were more common during the dry season compared to the parts of the year with average and above average rainfall (normal and wet season).

The spatial and temporal patterns of ASF outbreaks indicate that the virus can survive during periods without reports of ASF outbreaks. This could be partly because of underreporting or most importantly due to the transmission dynamics of the virus. The spreading of the infection through introduction of infected pigs, either during the incubation period or by persistently infected pigs, has been described as one of the most important transmission routes [16]. In addition, the fact that the virus is spread through both the sylvatic and the domestic cycles could be a reason for the observed patterns of outbreaks. The sylvatic cycle involves wild species of swine spreading the virus by soft ticks of the genus *Ornithodoros* [17]. In Africa the major host for the ASF virus is the warthog, but all wild species of swine in Africa can be silent carriers. The *Ornithodoros *ticks can survive for a long time and can harbour the virus for several years with only a gradual decrease of infectivity [17]. In commercial farms it is unlikely for the domestic pigs to come in contact with wild pigs and their ticks, but this is considered more common in traditional free-ranging systems [16]. A previous study carried out in Mubende district located in central Uganda suggested that free ranging and tethering could have an influence on the occurrence of ASF outbreaks [18]. In Uganda ASFV has been detected in the wild suids and in *Ornithodoros* ticks in Rakai district, and in the same study eight PCR-positive pigs were found with no known clinical disease [19]. In the intermediate cycle ASFV has been found in ticks collected in pig stys that have been empty of pigs for four years [20].

The domestic cycle involves domestic pigs spreading the virus to other domestic pigs through direct or indirect contact [17]. In the infected domestic pig the virus is shed in enormous amounts in all bodily secretions and excretions, tissues, and blood 24 to 48 hours before clinical symptoms are shown. Transmission through direct contact can occur up to 30 days after infection, whereas blood is infective for eight weeks [17] and in putrefied blood as long as 15 weeks [21]. Meat from infected pigs or contaminated pork products is another important source of infection due to the virus's long persistence in tissues [22]. The virus has been found in lymphoid tissues in domestic pigs for up to three–four months after infection [17]. The role of subclinical carriers has been widely described in several studies. Recovered pigs might remain persistently infected for 6 months and during this time act as a source of transmission to susceptible pigs [22]. In addition, symptomatic carrier animals play an important role in the persistence and dissemination of the disease in endemic areas [21]. In a recent study conducted in Gulu district in Northern Uganda, it was found out that in most rural areas, local slaughter places are small and poorly equipped and waste is directly accessible to other animals such as dogs or roaming pigs and that many pig owners sell their pigs as soon as they suspect ASF among them [23]. This could be one of the major modes of transmission and sources of outbreaks in other rural areas of Uganda.

In this study, a total of 201 outbreaks were reported in districts adjacent to the national parks representing 51.8% of the total outbreaks reported during the study period (2001–2012) with some national parks being more involved than others. This emphasizes the role of the wild suids in the epidemiology of ASF in Uganda. However, the results of this study cannot conclude on the dynamics of transfer of ASFV between domestic pigs and wild suids in Uganda. Eighty ASF outbreaks were reported in 18 districts along the international borders compared to 308 outbreaks reported in districts that did not share an international border. The number of ASF outbreaks varied between the different international borders, the highest being adjacent with DRC (31 outbreaks in eight districts) and Tanzania borders (26 outbreaks in 2 districts). Though the occurrence of ASF does not entirely depend on proximity to international borders, it is important to consider control of cross-border movements in the animal disease control programme in Uganda. The authors think that the porous nature along the two borders and the geography of the Eastern part of the DRC could be the cause of uncontrolled animal movements, hence the observed high number of ASF outbreaks. Notably, 26 outbreaks were reported in only 2 of the districts bordering Tanzania. A recent study has reported a genetic similarity in ASF disease outbreaks in Uganda and Kenya in 2003, 2006, and 2007 [24] emphasizing the role of cross-border animal movements in the epidemiology of ASF.

The perceptions of the DVOs on risk factors were highly suggestive that trade and restocking were the most important risk factors for the occurrence of ASF outbreaks. This important finding could not be obtained through analysis of spatial and temporal data. A combination of findings from the spatial and temporal studies and DVO-based analysis of risk factors and characteristics of ASF outbreaks in Uganda indicates that the risk is highest during the dry season. This could be as a result of lack of or limited feed resources by the farmers that lead to the sale of pigs or even due to the dynamics of pig movement in search of feeds. Twenty-seven of the 29 DVOs that responded reported that the ASF outbreaks in their districts originated from a neighbouring district indicating the role of animal movement in disease spread. Furthermore, this could probably be due to pig farmers' and traders' failure to adhere to quarantine measures when instituted during ASF outbreaks. One DVO (Kasese) reported the source of a previous outbreak to be across the border (DRC). This could be due to the porous nature of the border and lack of vigilance to enforce animal movement control. This could justify the high number of outbreaks along the DRC border compared to other international borders during the study.

The authors affirm that with regard to the validity of the data presented in this paper, the DVOs are well trained and experienced in animal disease surveillance and control measures, and it is highly likely that the information obtained through extracts from the regular reports to the central administration at MAAIF and through the questionnaire is too reliable. This study has found out that pig movement in the form of trade and restocking is an important factor in the transmission of ASF in Uganda. We strongly recommend that ASF control strategies should encompass a holistic value chain analysis. The government of Uganda through MAAIF should revise and enforce the restocking policy guidelines. More detailed and systematic studies should be undertaken to investigate further other specific risk factors and patterns of occurrence of ASF in Uganda.

## Figures and Tables

**Table 1:** Reported ASF outbreaks by year, month and region in Uganda (2001–2012).

Year	Region	Month												Total	
		J	F	M	A	M	J	J	A	S	O	N	D		
2001	C	—	1	3	—	—	5	4	4	4	2	5	5	33	(49)
	E	—	1	2	—	—	—	—	1	1	2	1	1	9	
	N	—	1	—	—	—	1	—	1	1	—	—	—	4	
	W	—	—	—	—	—	—	—	—	1	—	1	1	3	
	SW	—	—	—	—	—	—	—	—	—	—	—	—	0	
	WN	—	—	—	—	—	—	—	—	—	—	—	—	0	

2002	C	1	1	3	3	3	5	4	4	4	2	4	5	39	(59)
	E	1	1	2	2	1	—	—	1	1	2	2	1	14	
	N	—	1	—	—	—	1	—	1	1	—	—	—	4	
	W	—	—	—	—	—	—	—	1	—	—	—	1	2	
	SW	—	—	—	—	—	—	—	—	—	—	—	—	0	
	WN	—	—	—	—	—	—	—	—	—	—	—	—	0	

2003	C	4	3	2	2	1	3	1	—	4	—	3	2	25	(64)
	E	3	3	3	2	1	—	2	2	2	1	4	2	25	
	N	—	1	—	1	1	1	—	—	—	—	—	1	5	
	W	—	—	1	1	1	2	2	—	—	—	—	—	7	
	SW	—	—	1	1	—	—	—	—	—	—	—	—	2	
	WN	—	—	—	—	—	—	—	—	—	—	—	—	0	

2004	C	1	2	1	2	3	4	2	2	2	1	1	4	25	(57)
	E	4	3	4	2	2	3	1	3	—	—	—	—	22	
	N	—	1	1	—	—	—	—	—	—	1	2	—	5	
	W	—	—	—	—	—	—	1	—	—	—	1	—	2	
	SW	—	—	—	—	—	—	1	—	1	1	—	—	3	
	WN	—	—	—	—	—	—	—	—	—	—	—	—	0	

2005	C	3	2	—	—	—	—	—	—	—	—	—	—	5	(17)
	E	1	—	2	3	—	—	—	—	—	—	—	—	6	
	N	—	1	2	1	—	—	—	1	—	—	—	—	5	
	W	—	—	—	—	—	—	—	—	—	—	—	—	0	
	SW	1	—	—	—	—	—	—	—	—	—	—	—	1	
	WN	—	—	—	—	—	—	—	—	—	—	—	—	0	

2006	C	—	—	—	—	—	—	—	—	—	—	—	—	0	(14)
	E	1	1	2	1	1	—	—	—	—	—	—	1	7	
	N	—	—	—	—	—	—	—	—	—	—	1	1	2	
	W	—	—	—	—	—	—	—	—	—	—	—	—	0	
	SW	—	—	—	—	—	—	—	—	—	—	—	—	0	
	WN	1	1	—	—	—	—	—	1	—	1	1	—	5	

2007	C	—	—	—	—	—	1	2	—	—	—	—	1	4	(9)
	E	—	—	—	1	—	—	—	1	1	—	—	—	3	
	N	—	2	—	—	—	—	—	—	—	—	—	—	2	
	W	—	—	—	—	—	—	—	—	—	—	—	—	0	
	SW	—	—	—	—	—	—	—	—	—	—	—	—	0	
	WN	—	—	—	—	—	—	—	—	—	—	—	—	0	

2008	C	—	2	1	1	2	—	1	—	—	1	1	1	10	(20)
	E	—	1	1	—	—	—	—	—	—	—	1	1	4	
	N	—	—	—	—	—	—	—	—	—	—	—	—	0	
	W	—	—	2	1	—	—	—	—	—	—	—	—	3	
	SW	—	—	—	—	—	—	—	—	—	1	1	1	3	
	WN	—	—	—	—	—	—	—	—	—	—	—	—	0	

2009	C	—	—	—	—	—	—	—	—	—	1	—	—	1	(6)
	E	1	—	1	1	1	—	—	—	—	—	—	—	4	
	N	—	—	—	—	—	—	—	—	—	—	—	—	0	
	W	—	—	—	—	—	—	—	—	—	—	—	—	0	
	SW	1	—	—	—	—	—	—	—	—	—	—	—	1	
	WN	—	—	—	—	—	—	—	—	—	—	—	—	1	

2010	C	—	—	—	—	—	—	1	—	—	—	1	—	2	(9)
	E	—	—	—	1	—	—	1	1	—	—	1	—	4	
	N	—	—	—	—	—	1	—	—	1	—	—	—	2	
	W	—	—	—	—	—	—	—	—	—	—	—	—	0	
	SW	—	—	—	—	—	1	—	—	—	—	—	—	1	
	WN	—	—	—	—	—	—	—	—	—	—	—	—	0	

2011	C	4	2	1	3	2	7	2	2	1	2	1	2	29	(68)
	E	1	—	—	—	1	—	—	1	—	—	—	1	4	
	N	4	8	2	3	—	1	1	1	—	—	1	1	22	
	W	—	1	1	—	—	1	—	—	—	—	—	—	3	
	SW	—	—	—	—	—	—	—	1	—	—	—	—	1	
	WN	3	—	—	—	—	—	—	1	1	1	1	2	9	

2012	C	—	2	1	—	—	1	—	—	—	—	—	—	4	(16)
	E	—	—	—	—	—	—	—	—	—	—	—	—	0	
	N	3	1	2	—	—	1	1	1	1	—	—	—	10	
	W	—	—	—	—	—	1	—	—	—	—	—	—	1	
	SW	—	—	—	—	—	—	—	—	—	—	—	—	0	
	WN	—	—	1	—	—	—	—	—	—	—	—	—	1	

	Total	38	43	42	32	20	40	27	32	26	19	34	35		(388)

C: central; E: eastern; N: northern; W: western; SW: southwestern; WN: west nile

Dashes (—) represent periods when there were no newly reported ASF outbreaks.

**Table 2:** Assessment of patterns of occurrence of ASF outbreaks and the distribution of rainfall in selected districts in Uganda (2001–2008).

Region	District	Mean annual rainfall (mm)	ASF outbreaks by rainfall (mm)			Total number of outbreaks
			Above average^a^	Average^b^	Below average^c^	
N	Apac	1489	4	0	9	13
N	Kitgum	1343	1	0	3	4
W	Masindi	1317	2	3	9	14
C	Wakiso	1216	7	1	6	14
C	Nakasongola	1046	0	1	4	5
C	Rakai	987	2	5	7	14
E	Kamuli	1387	3	5	8	16
SW	Kasese	884	0	1	4	5
WN	Moyo	1459	0	0	1	1

Total			19	16	51	86

Monthly rainfall (mm) was evaluated as average (normal season), below average (dry season), or above average (wet season) based on long-term mean. Mean annual rainfall values (mm) represent the distribution across selected weather stations and regions.

^a^Wet season.

^b^Neither dry nor wet season.

^c^Dry season.

**Table 3:** Regional reported ASF outbreaks 2001–2012.

Region	Number of outbreaks (%)
Central	181 **(46.6)**
Eastern	100 **(25.8)**
Northern	60 **(15.5)**
Southwestern	12 **(3.1)**
Western	23 **(5.9)**
West Nile	12 **(3.1)**

Total	388

**Table 4:** Occurrence of ASF outbreaks within the districts adjacent to the national parks (2001–2012).

National parks	Year of ASF occurrence												Total
	2001	2002	2003	2004	2005	2006	2007	2008	2009	2010	2011	2012	
BINP	—	—	—	2	1	—	—	—	—	—	—	—	3
KINP	—	—	2	5	—	—	—	—	—	—	3	—	10
KVNP	1	1	—	—	2	2	1	—	—	—	4	2	13
LMNP	4	4	3	—	—	—	—	1	—	—	4	1	17
MENP	5	7	16	10	4	2	3	4	1	3	3	—	58
MFNP	6	5	13	6	5	6	2	6	1	4	29	8	91
QENP	—	—	2	1	—	—	—	3	1	1	1	—	9

BINP: Bwindi impenetrable national park, KINP: Kibale national park, KVNP: Kidepo valley national park, LMNP: Lake Mburo national park, MENP: Mount Elgon national park, MFNP: Murchison Falls national park, QENP: Queen Elizabeth national park.

**Table 5:** Perceived characteristics of ASF occurrence and control in Uganda.

Perceived variables	Parameters	Response (numbers/proportion)		
		Yes	No	NA
Cases of pig deaths or sickness		29 (1.00)	0 (0.00)	0 (0.00)

Percentage of the pig population affected	<5%	7 (0.24)	22 (0.76)	0 (0.00)
	5–15%	8 (0.28)	21 (0.72)	0 (0.00)
	20–25%	6 (0.21)	23 (0.79)	0 (0.00)
	Over 50%	8 (0.28)	21 (0.72)	0 (0.00)

Rating of ASF as an important disease in relation to other diseases	ASF (rank = 3.76)	24 (0.83)	5 (0.17)	0 (0.00)
	Helminthiasis/worms (rank = 2.21)	29 (1.00)	0 (0.00)	0 (0.00)
	Malnutrition (rank = 8.76)	11 (0.38)	18 (0.62)	0 (0.00)
	Piglet anaemia (rank = 11.72)	4 (0.14)	25 (0.86)	0 (0.00)
	Cysticercosis (rank = 12.62)	1 (0.03)	28 (0.97)	0 (0.00)
	Mange/Ectoparasites (rank = 6.41)	18 (0.64)	11 (0.36)	0 (0.00)
	Swine Dysentery (rank = 12.28)	4 (0.14)	25 (0.86)	0 (0.00)
	Swine erysipelas (rank = 12.31)	1 (0.03)	28 (0.97)	0 (0.00)
	Abortions (rank = 12.28)	2 (0.07)	27 (0.93)	0 (0.00)
	Pneumonia (rank = 12.31)	1 (0.03)	28 (0.97)	0 (0.00)
	Trypanosomosis (rank = 11.79)	4 (0.14)	25 (0.86)	0 (0.00)
	Lameness (rank = 12.69)	1 (0.03)	28 (0.97)	0 (0.00)
	Ticks (rank = 12.66)	1 (0.03)	28 (0.97)	0 (0.00)

Most recent incident of ASF outbreak	6 months ago	10 (0.35)	19 (0.65)	0 (0.00)
	Still ongoing	9 (0.31)	20 (0.69)	0 (0.00)
	1 year ago	5 (0.17)	24 (0.83)	0 (0.00)
	More than 1 year ago	5 (0.17)	24 (0.83)	0 (0.00)
	Shivering	1 (0.03)	28 (0.97)	0 (0.00)

Seasons when incidence is most frequent	Dry seasons	14 (0.48)	8 (0.28)	7 (0.24)
	Wet seasons	3 (0.10)	19 (0.66)	7 (0.24)
	Every after 5 years	1 (0.03)	21 (0.72)	7 (0.24)
	Every after 2 years	1 (0.03)	21 (0.72)	7 (0.24)
	Throughout the year	4 (0.14)	18 (0.62)	7 (0.24)

Months when ASF is frequent	January–March	3 (0.10)	26 (0.90)	0 (0.00)
	April–June	0 (0.00)	29 (1.00)	0 (0.00)
	July–September	2 (0.07)	27 (0.93)	0 (0.00)
	October–December	2 (0.07)	27 (0.93)	0 (0.00)
	January–June	1 (0.03)	28 (0.97)	0 (0.00)
	Throughout the year	8 (0.28)	21 (0.72)	0 (0.00)
	January–March, July–September	3 (0.10)	26 (0.90)	0 (0.00)
	January–March, October–December	2 (0.07)	27 (0.93)	0 (0.00)
	July–December	4 (0.14)	25 (0.84)	0 (0.00)

Actions taken to control ASF	Quarantine	23 (0.79)	5 (0.21)	1 (0.03)
	Slaughter	7 (0.24)	21 (0.76)	1 (0.03)
	Burial of the dead	6 (0.21)	22 (0.79)	1 (0.03)
	Avoid immediate restocking	1 (0.03)	27 (0.97)	1 (0.03)
	Community sensitization	8 (0.28)	20 (0.72)	1 (0.03)
	Disinfection	1 (0.03)	27 (0.97)	1 (0.03)
